# Imaging findings and classification of the common and uncommon male breast diseases

**DOI:** 10.1186/s13244-019-0834-3

**Published:** 2020-02-18

**Authors:** Ömer Önder, Aynur Azizova, Gamze Durhan, Funda Dinç Elibol, Meltem Gülsün Akpınar, Figen Demirkazık

**Affiliations:** 1 Department of Radiology, School of Medicine, Hacettepe University, 06100 Ankara, Turkey; 2 Department of Radiology, School of Medicine, Muğla Sıtkı Koçman University, 48000 Muğla, Turkey

**Keywords:** Male breast lesion, Classification, Ultrasound, Mammography, Imaging findings

## Abstract

Male breast hosts various pathological conditions just like “female breast.” However, histo-anatomical diversities with female breast lead to many differences regarding the frequency and presentation of diseases, the radiologic appearance of lesions, the diagnostic algorithm, and malignity features.

Radiological modalities may play an important role in evaluating male breast lesions. Although some imaging findings are non-specific, having knowledge of certain imaging characteristics and radiologic patterns is the key to reduce the number of differential diagnoses or to reach an accurate diagnosis.

Male breast imaging is mostly based on physical examination and is required for the complaints of palpable mass, breast enlargement, tenderness, nipple discharge, and nipple-skin changes. The majority of the male breast lumps are benign and the most common reason is gynecomastia. Although it is difficult to exclude malignancy in some cases, gynecomastia often has distinguishable imaging features. Pseudogynecomastia is another differential diagnosis that may be confused with gynecomastia. The distinction is important for the treatment plan.

Apart from gynecomastia, other male breast lesions form a highly heterogeneous group and can be classified based on “Tissue origin,” “Histopathological type and behavior,” and “Radiologic features” for both simplification and comprehensive understanding.

This article mainly focuses on emphasizing the results of basic histo-anatomical differences of male and female breasts, classifying male breast lesions, covering the spectrum of male breast diseases, and assisting radiologists in recognizing the imaging findings, in interpreting them through a holistic approach, in making a differential diagnosis, and in being a part of proper patient management.

## Key points

- Male and female breasts have certain histo-anatomical differences that can affect the spectrum of diseases.
- The majority of male breast lumps are benign and the most common reason is gynecomastia. Pseudogynecomastia and malignancy are the most common differential diagnoses.
- Male breast imaging is mostly based on “physical examination,” and radiological modalities can play an important role.
- Imaging of the male breast has many aspects regarding not only medical and scientific but also social concerns that can lead to poor patient compliance and follow-up losses.
- Male breast lesions form a highly heterogeneous group and could be classified based on various parameters for comprehensive understanding.

## Introduction

The male breast is located between the 2nd–6th ribs craniocaudally and the midaxillary line–sternum lateromedially, like female breast. Although they share similar locations, there are several differences between them, which concern the developmental process and histo-anatomical structures [1].

The mammary glands of both sexes are identical at birth. During peripubertal period, in the female breast, ductal proliferation, branching, and growth are stimulated due to estrogen. As for stromal development and terminal ductal-lobular unit (TDLU) maturation, they are seen due to progesterone. However, involution and ductal atrophy occur in the male breast due to significant increase in testosterone levels [2].

**Fig. 1:**
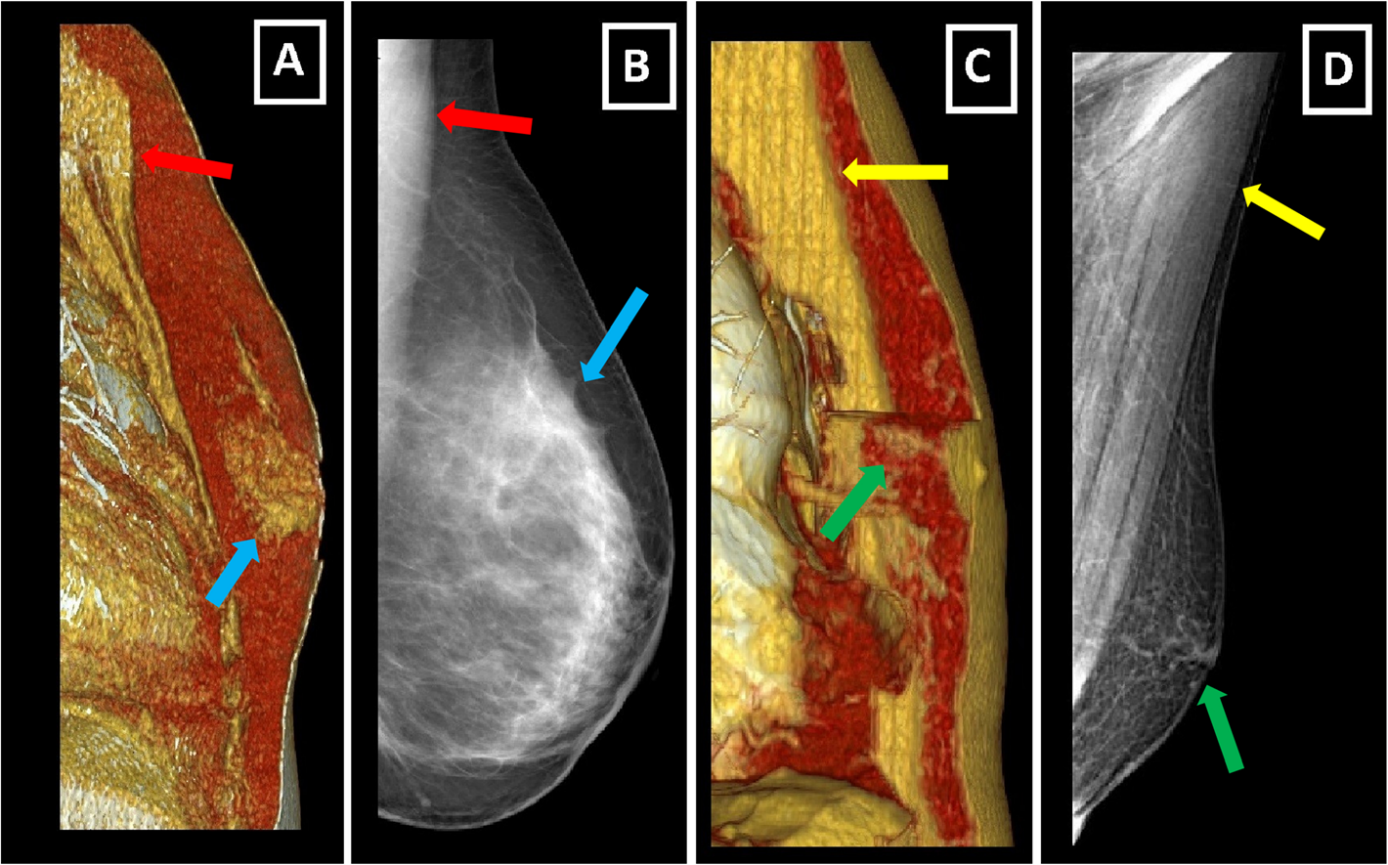
Differences between female and male breast. **a**, **b** Oblique sagittal VRT image and MLO mammography image of normal female breast. Note the well-developed fibroglandular structures (blue arrows) and smaller pectoral muscles (red arrows). **c**, **d** Oblique sagittal VRT image and MLO mammography image of normal male breast. Note the rudimentary structure of male breast with involuted fibroglandular tissue, small nipple-areolar complex (green arrows), and prominent pectoral muscles (yellow arrows)

In contrast to the female breast, Cooper ligaments are absent, ductal system is involuted, TDLU development is rare, stromal system is smaller in size and pectoralis muscles are more prominent in the male breast [3] (Fig. 1).

**Fig. 2:**
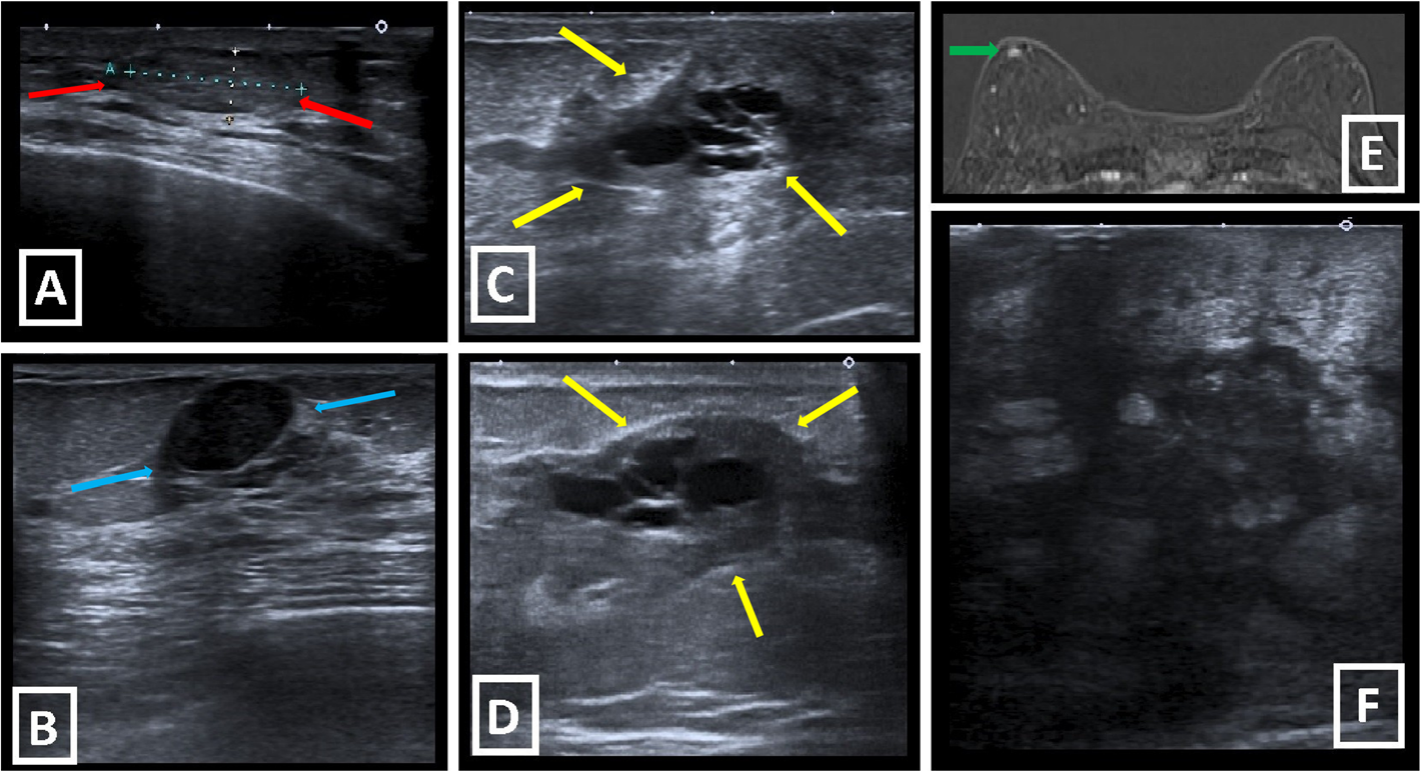
Uncommon male breast lesions due to fibroepithelial or lobular origin. **a** Fibroadenoma is seen as well-defined, hypoechoic, solid lesion with posterior acoustic enhancement in US (red arrows). **b** Circumscribed, hypoechoic, solid lesion with slight posterior acoustic shadowing is diagnosed as “Adenosis” after biopsy (blue arrows). **c**, **d** Fibrocystic changes with apocrine metaplasia. US shows multiseptated cystic structures within retroareolar heterogeneous hypoechoic area in a 59-year-old male patient with palpable mass (yellow arrows). **e** After detecting right retroareolar ductal ectasia with peripheral hypoechoic heterogeneous area in US (not shown), subtraction image of dynamic MRI of 72-year-old male patient shows retroareolar well-defined enhancing focus which corresponds to the defined lesion. Biopsy reveals the diagnosis of “Fibroadenomatoid changes”. **f** Large, heterogeneous, ill-defined, hypoechoic mass with subcutaneous edema and pectoral muscle invasion (not shown) is diagnosed as ILC

Involution of ductal system causes decreased ductal branching, so ductal malignancies are rarely encountered in males and are located in close proximity to nipple-areolar complex when they exist. Breast cancer incidence is low in the male population, so there is no need for routine screening. Due to delay in diagnosis, male breast cancer is detected at a more advanced stage when compared with female breast cancer. At the time of diagnosis, axillary lymphadenopathy (LAP) involvement is at nearly 50%, which is more frequent than female breast cancer, and secondary signs, such as skin thickening, ulceration, and increased trabeculations, appear earlier. Moreover, microcalcifications, which may show ductal involvement, are seen less likely in male breast cancers, probably because of involuted ductal structure. Other than that, fibroepithelial (biphasic, i.e., fibroadenoma, phylloides tumor) and lobular (invasive lobular carcinoma (ILC), lobular carcinoma in situ (LCIS), fibrocystic changes, adenosis) pathologies are uncommon due to rare development of TDLU [2–6] (Fig. 2).

**Fig. 3:**
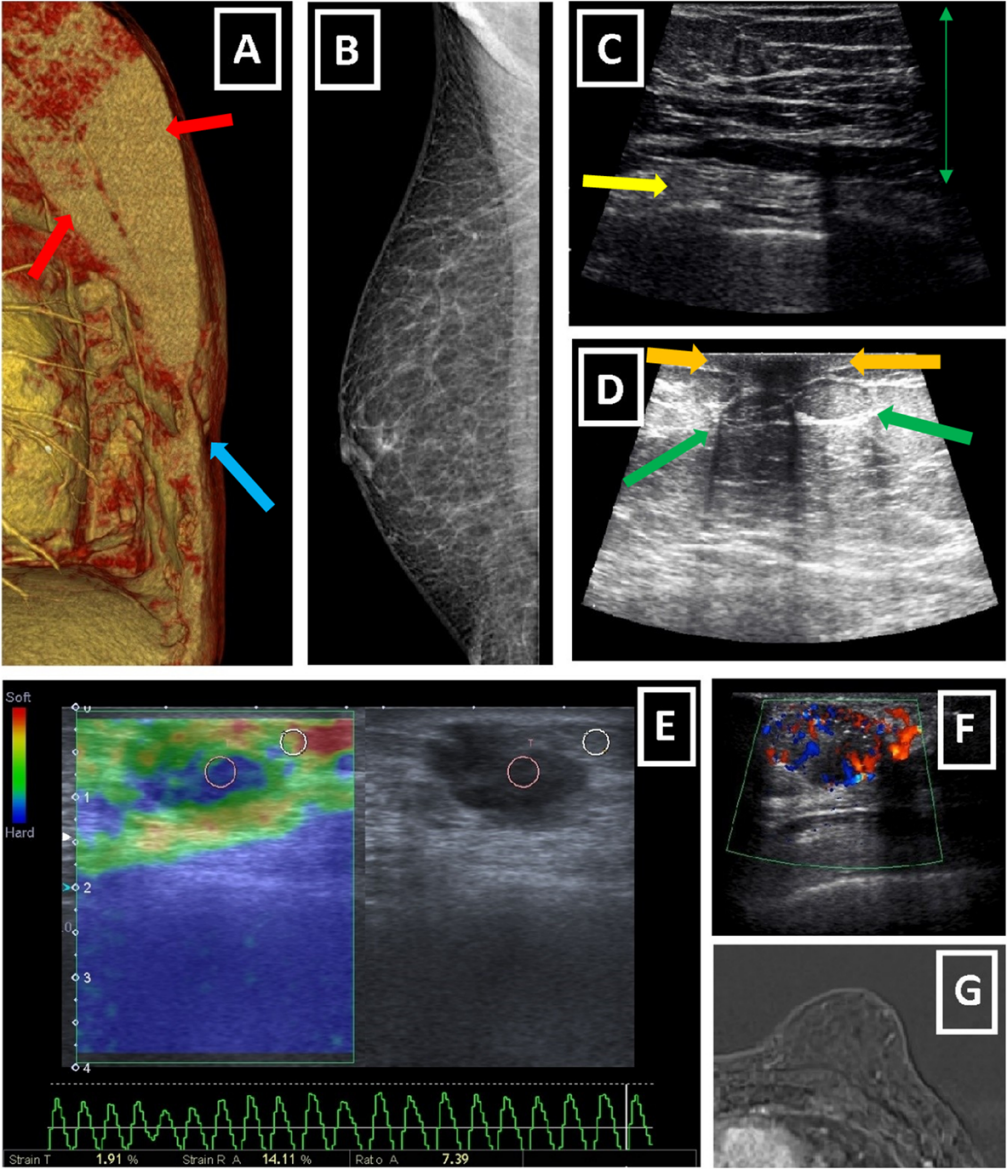
Imaging modalities used for male breast diseases. **a** Oblique sagittal VRT reformat of a CT scan obtained for an irrelevant purpose shows hypertrophic pectoralis major and minor muscles (red arrows) and retroareolar scarce fat tissue (blue arrow). Both can cause technical and diagnostic difficulties for mammography. Although it can show masses incidentally, CT is not a routine imaging modality for male breast. **b** MLO mammogram of right male breast shows rudimentary fibroglandular tissue replaced with increased amount of radiolucent adipose tissue. **c**, **d** US images of male breast. Green arrows show subcutaneous fat tissue characterized by isoechoic fat lobules. Yellow arrow (**c**) represents intercostal muscles and orange arrow (**d**) corresponds to small nipple-areola complex with no associated fibroglandular tissue. **e** Sonoelastographic evaluation of ill-defined hypoechoic male breast mass reveals elevated tissue stiffness with considerably increased strain ratio of 7.39 and suggests malignity. Pathologic examination confirms the diagnosis of IDC. **f** Color mode of Doppler US shows markedly increased peripheral and internal vascularity of irregular hypoechoic male breast mass which is diagnosed as IDC later. **g** Subtraction image of dynamic breast MRI of a male patient with known pseudogynecomastia shows enlarged left male breast without apparent enhancing lesion

Male breast as a rudimentary structure consists of subcutaneous adipose tissue, remnant ductal tissue, and small nipple-areolar complex (Fig. 1). Keeping structure and components of the male breast in mind facilitates radiologic assessment. Mammography, ultrasound (US), and rarely magnetic resonance imaging (MRI) are the main imaging modalities (Fig. 3).

In mammography, a normal male breast is composed of homogenously radiolucent fat tissue and prominent radio-opaque pectoralis muscle. Small volume of male breast may cause technical difficulties. Moreover, prominent pectoralis muscle may obscure suspicious lesions [1, 4, 6, 7].

US imaging of the male breast has similar characteristics to that of the female breast. Isoechoic fat lobules corresponding to subcutaneous adipose tissue is a major finding of normal male breast seen in US. US can be very helpful for evaluating nipple-mass relationship and axillary nodal involvement. In case of suspicious lesions, vascularity assessment can be done using the Doppler mode. Sonoelastography may be used as auxiliary modality, although not on a routine basis [6–9].

MRI of male breast has limited use. According to literature, it can be used for evaluation of chest wall involvement of malignity, post-operative residual disease, and neoadjuvant chemotherapy response [10, 11].

Imaging of the male breast has an impact not only on medical and the scientific field but also on social issues. It could turn out to be somewhat problematic due to the social prejudices it is often associated with this. Social stigma related to male patient undergoing mammography or breast US within breast imaging centers, which are mostly used by female patients, may cause poor patient compliance and decreases in follow-up attendance [12].

There is no widely accepted consensus for diagnostic imaging algorithm of the male breast lesions. According to Mustapha et al., mammography is the first-line suggested imaging modality in case of suspicious physical examination. US is the next recommended step when mammography findings are inconclusive [13]. On the other hand, ACR appropriateness criteria suggest an age limit of 25 years old for first-line imaging modality. US is recommended as first-line imaging below 25 years old, whereas mammography is suggested after 25 years old. According to ACR, further imaging is not required when clinical findings are consistent with gynecomastia or pseudogynecomastia. However, mammography is proposed as a first step, if suspicion of cancer is high based on clinical findings [14].

Biopsy is required for pathological diagnosis when imaging modalities are equivocal. For male breast, US guided biopsy is preferred, as stereotactic biopsies are technically difficult due to smaller breast sizes [3, 6, 15].

### Gynecomastia, types, and differential diagnosis

**Fig. 4:**
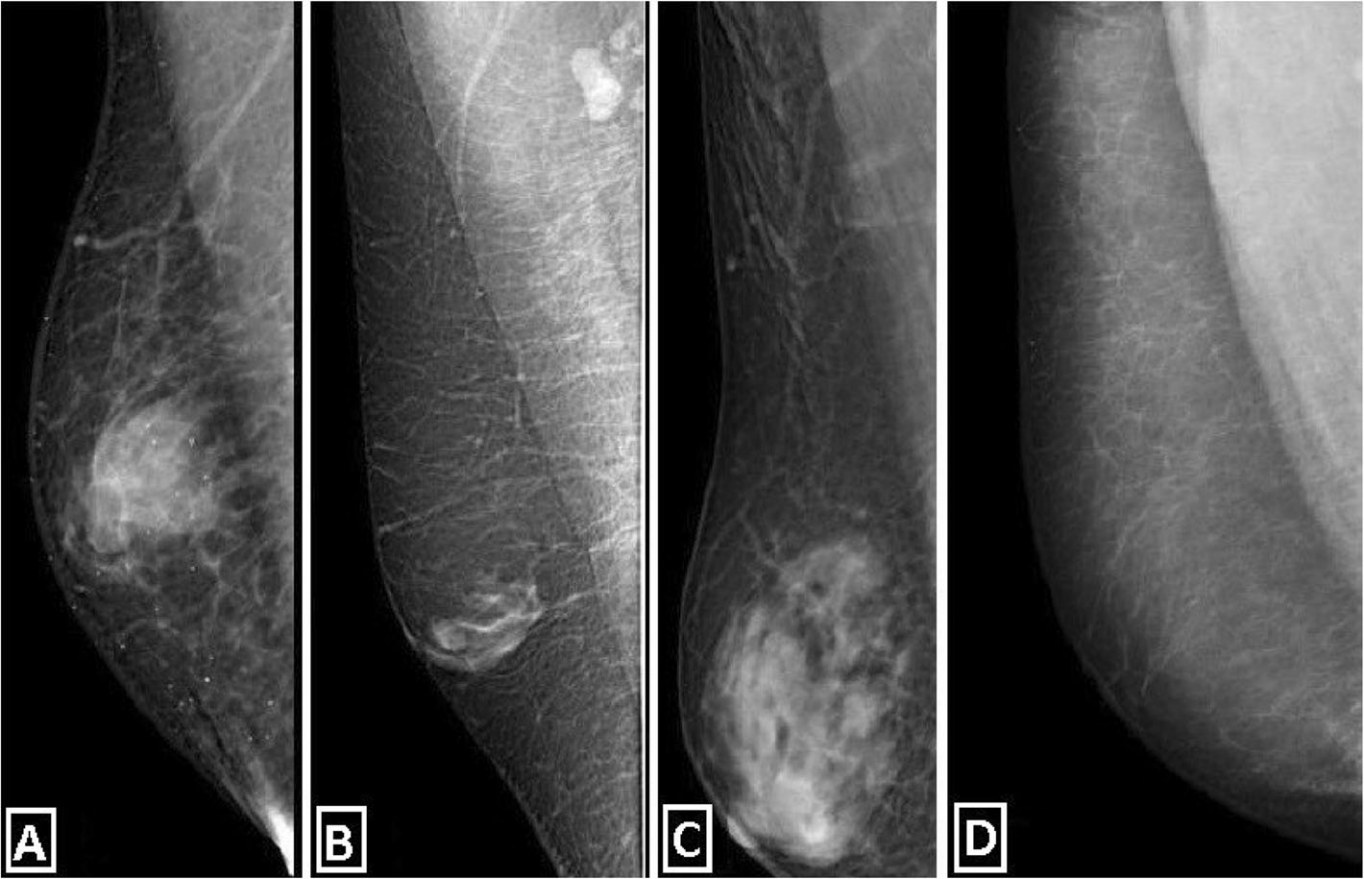
Medio-lateral oblique mammograms of gynecomastia types and pseudogynecomastia as common causes of the male breast lump. Nodular (**a**), dendritic (**b**), and diffuse glandular (**c**) types of gynecomastia are demonstrated. Pseudogynecomastia which refers to breast enlargement in men primarily due to fatty tissue without associated fibroglandular tissue (**d**)

The majority of male breast lumps are benign, and the most common reason is gynecomastia. Gynecomastia is formed by the proliferation of ductal and stromal elements, mostly seen during pubertal period or senescence, either presented as unilateral or bilateral breast mass along with breast enlargement and/or focal pain. Etiology of gynecomastia contains a wide-range spectrum including physiologic, endocrinologic, metabolic, neoplastic, and drug-induced causes. There are three characteristic patterns related to gynecomastia which are nodular, dendritic, and diffuse glandular forms [3, 6, 7] (Table 1, Fig. 4).

**Table 1:**
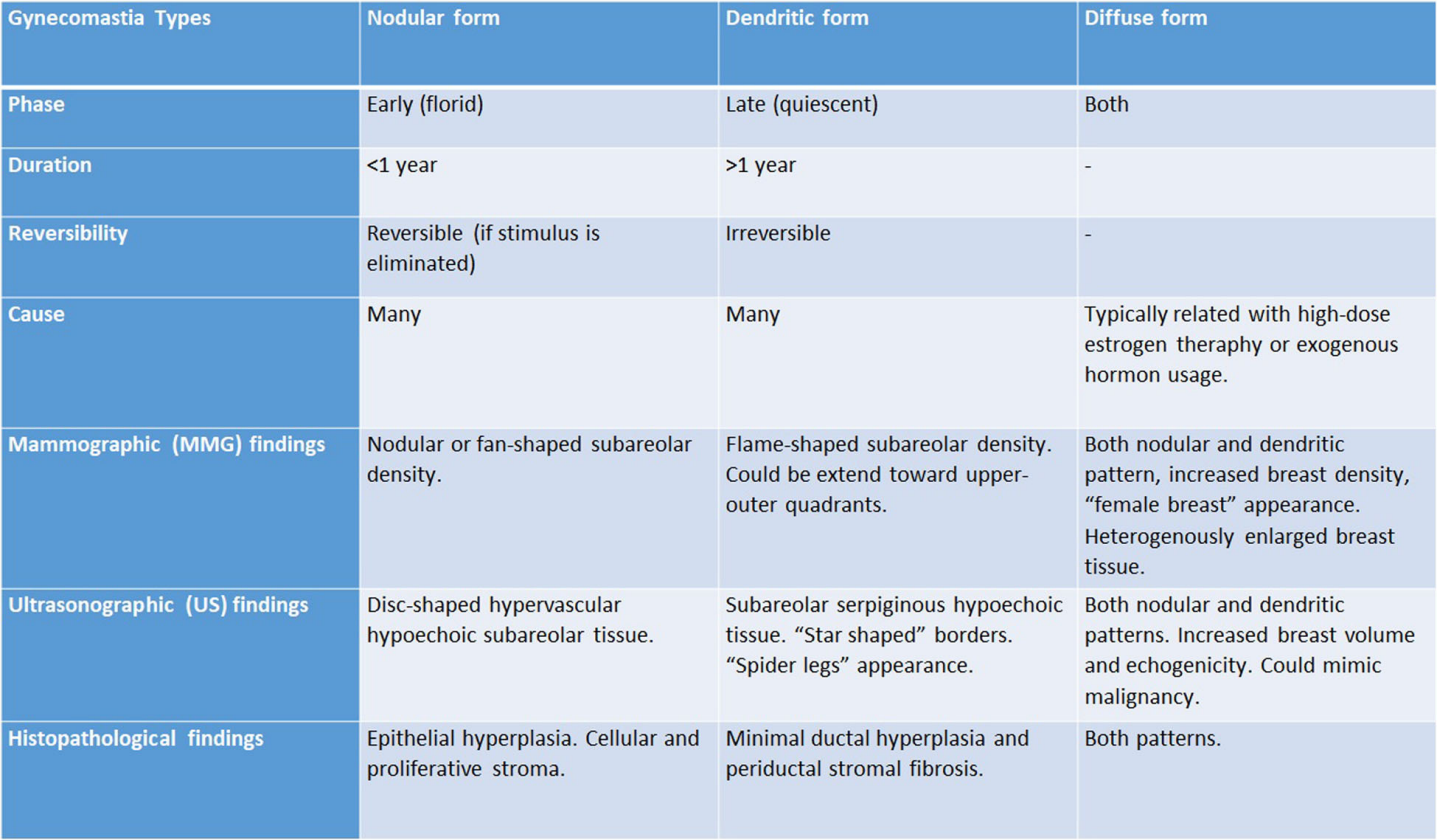
Gynecomastia types

The nodular form is characterized by prominent ductal hyperplasia and cellular/proliferative stroma. Generally, it is the sign of the early (florid) phase corresponding to a duration of less than 1 year. Nodular gynecomastia is accepted as a reversible pathology if underlying cause(s) is eliminated. It is seen as nodular or fan-shaped subareolar opacity in mammography and disk-shaped, hypervascular, hypoechoic subareolar tissue in US [6, 7, 15].

The dendritic form corresponds to periductal stromal fibrosis and minimal ductal hyperplasia. It is known as the late (quiescent) phase of a duration exceeding 1 year. It is irreversible due to chronic changes and fibrosis. Flame-shaped subareolar opacity which may extend toward upper-outer quadrants is an expected mammographic finding. Subareolar serpiginous tissue with “star-shaped” borders and “spider leg” appearance are the main characteristic findings of US [3, 6, 7, 15, 16].

The diffuse form has the properties of both early and late phases. It is typically related to high-dose estrogen therapy or exogenous hormone usage. Transgender male breasts usually show the diffuse pattern. Heterogeneously enlarged breast tissue with female breast appearance is seen in mammography. The main anatomic difference between diffuse gynecomastia and female breast is the absence of Cooper ligaments. In US, increased breast volume and echogenicity are present which may be falsely interpreted as malignity [3, 6, 7, 15, 16].

The most common differential diagnoses of gynecomastia are pseudogynecomastia and malignancy. Pseudogynecomastia refers to diffuse adipose tissue proliferation without fibroglandular development or discrete mass in male breast, which can be unilateral or bilateral. It is mostly seen in overweight–obese individual or with those with Neurofibromatosis type 1 (NF-1) history. Differentiation from gynecomastia could be important for the treatment plan. Liposuction alone is mostly sufficient for the treatment of pseudogynecomastia. On the other hand, surgical excision of glandular tissue can be required for a satisfactory treatment of gynecomastia [3, 4, 6, 17] (Table 2, Figs. 3b and 4d).

**Table 2:**
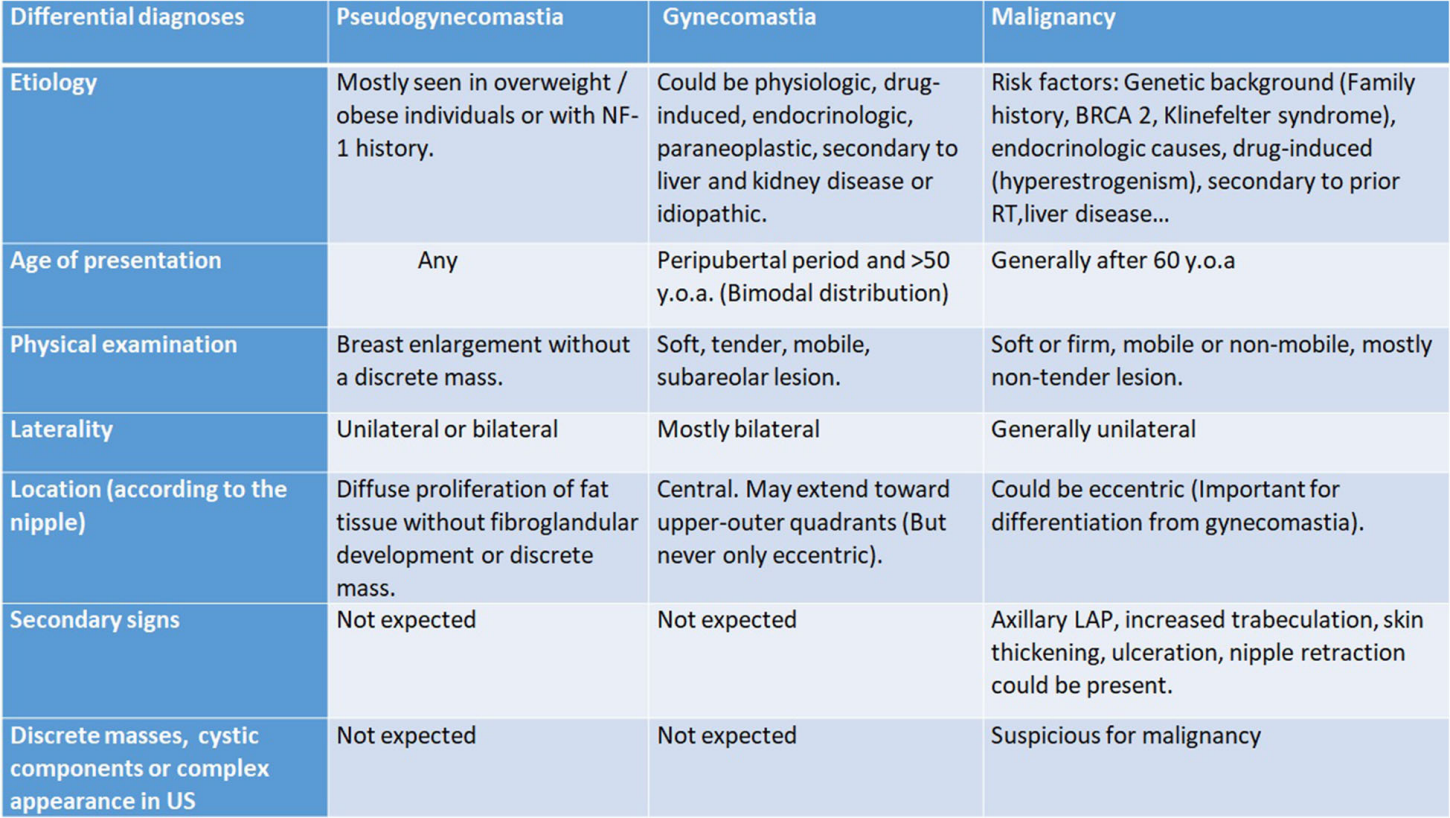
Differential diagnosis of gynecomastia

**Fig. 5:**
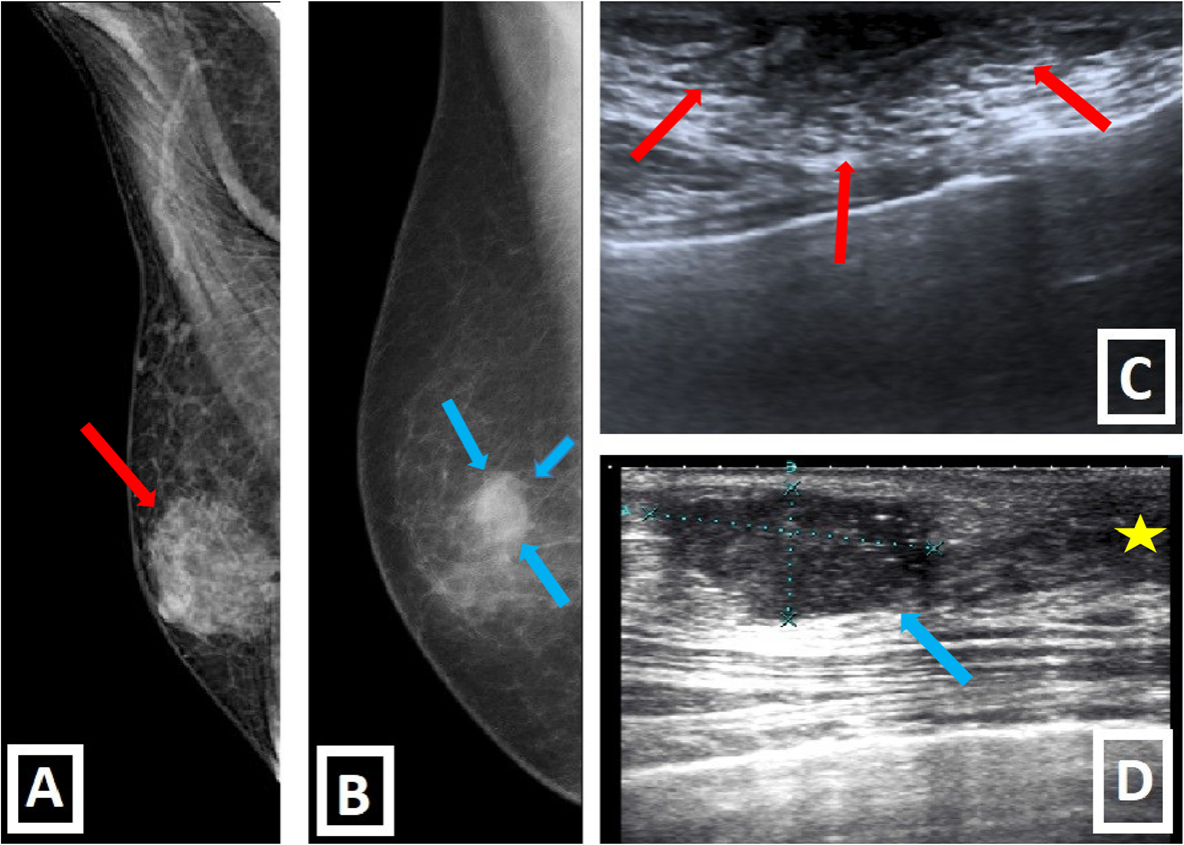
Gynecomastia vs. Primary breast malignity. **a**–**c** Fan-shaped central subareolar opacity, in mammography, and disk-shaped hypoechoic retroareolar tissue, in US (red arrows), with bilateral involvement (not shown) are typical imaging findings of gynecomastia (nodular form). **b**–**d** IDC of male breast. Irregular lesion with slightly eccentric location according to the nipple and higher density than accompanying background gynecomastia is seen in mammography. US shows ill-defined, hypoechoic, solid mass located outside of the nipple-areolar complex (yellow star) with unilateral involvement (blue arrows)

The distinction of malignancy and gynecomastia is critical because further intervention can be avoided when imaging findings are consistent with gynecomastia. Unfortunately this distinction is not always clear-cut. Although gynecomastia often has characteristic clinical and radiological features which help distinguish it from malignancy, it may be difficult to exclude malignancy in some cases (Table 2, Fig. 5).

While gynecomastia has the abovementioned risk factors, the risk factors of malignancy are different such as genetic background (family history, BRCA2 mutation, Klinefelter syndrome), liver disease, prior radiotherapy history, endocrinologic, or drug-related causes. Gynecomastia has bimodal age distribution involving the peripubertal period and the period after 50 years. On the other hand, malignancy usually occurs after 60 years of age. Bilateral involvement is usually expected in gynecomastia while unilateral involvement is usually expected in malignancy. Central involvement is a rule for gynecomastia. Although extension to the upper-outer quadrants may be encountered, pure eccentric lesions are not expected in gynecomastia. In the malignancy, lesions can be eccentric, and it is important for differentiation from gynecomastia. Gynecomastia mostly present as soft, tender, mobile subareolar lesion. On the other hand, malignancy can be encountered as soft or firm, mobile or non-mobile, and mostly non-tender lesion. Accompanying secondary signs including skin thickening, ulceration, nipple retraction, increased breast trabeculation, and axillary LAPs, favors malignancy. Discrete masses, cystic components, and complex sonographic appearances also suggest malignant pathology [3, 6, 7, 15].

### Classification and spectrum of disease

Apart from gynecomastia, male breast lesions form a highly heterogenous spectrum which will be discussed elaborately. They can be grouped based on various parameters, such as “Tissue origin,” “Histopathological type and behavior,” and “Radiologic features,” for simplification, comprehensive understanding, and reasonable radio-pathologic integration.

#### Tissue origin

**Fig. 6:**
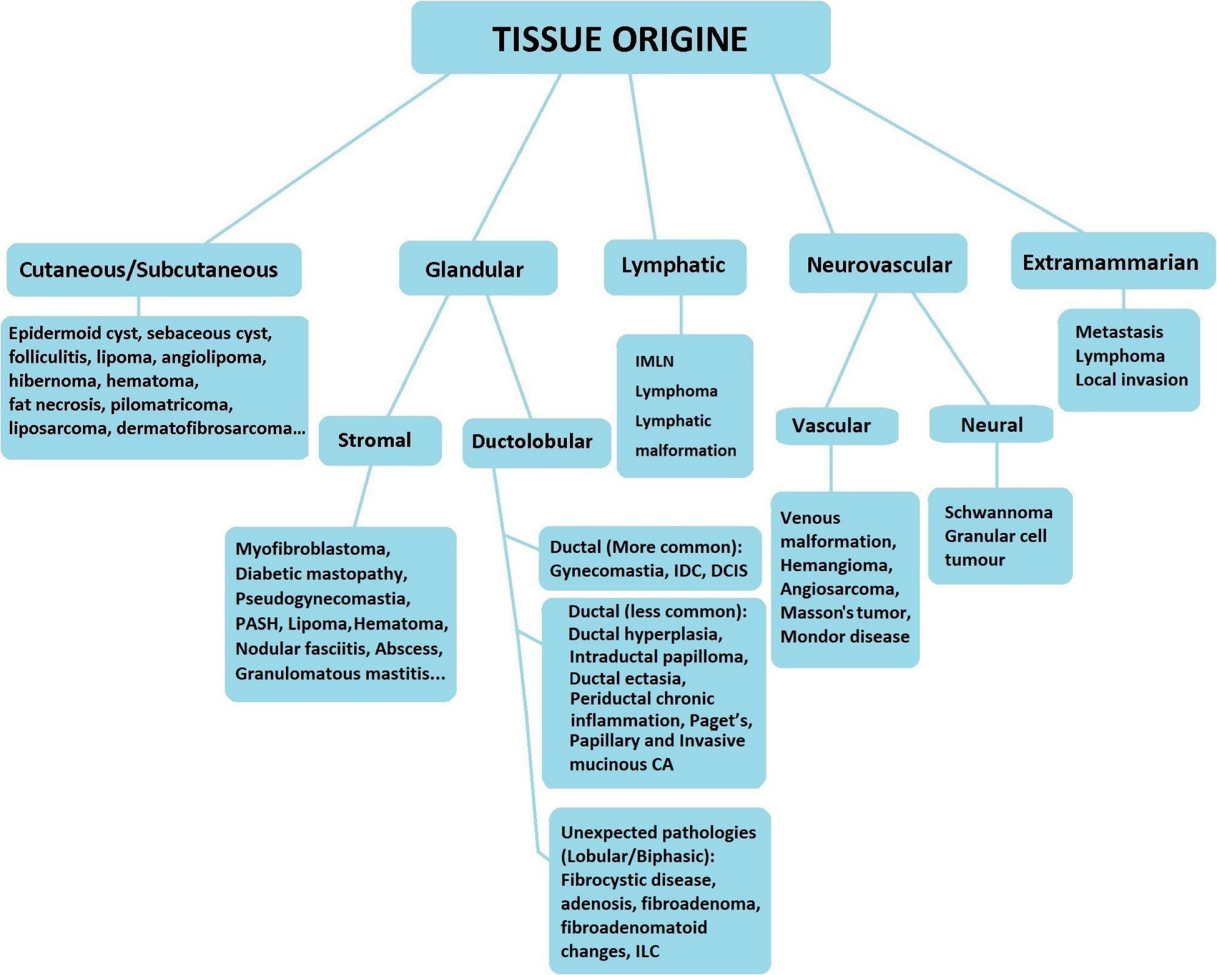
Classification based on “Tissue origin”

Regarding tissue origin, male breast lesions may develop from cutaneous/subcutaneous tissues, glandular/stromal breast tissues, neurovascular tissues, lymphatic tissues, and extramammarian lesions (Fig. 6). Breast lesions from extramammarian origin are further classified, mainly, as solid organ metastases (prostate, lung, gastric cancers), and hematological malignancy involvement (lymphoma, leukemia, plasmacytoma). Local invasion of malignancies in close proximity to the breast, such as lung cancer, is also a probability [1, 15, 18].

#### Histopathological type and behavior

**Fig. 7:**
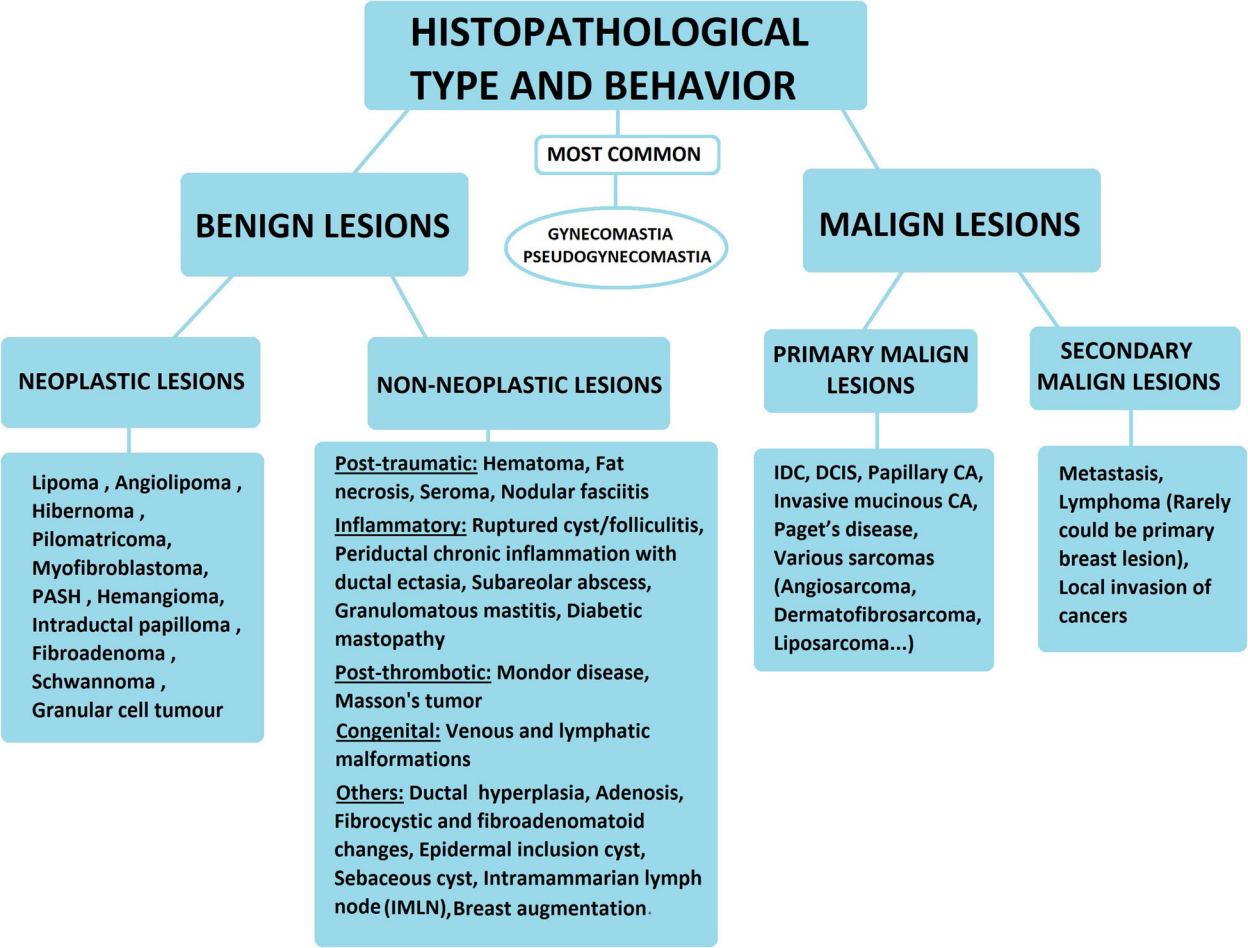
Classification based on “Histopathological type and behavior”

According to histopathological type and behavior we can divide male breast lesions into two main categories as “benign” and “malign.” Benign lesions are subdivided into two parts as “neoplastic” and “non-neoplastic.” Non-neoplastic benign lesions are further classified according to their etiologies. Malign lesions are classified as “primary” and “secondary” according to their origins (Fig. 7) [3, 6, 7].

#### Radiological features

**Fig. 8:**
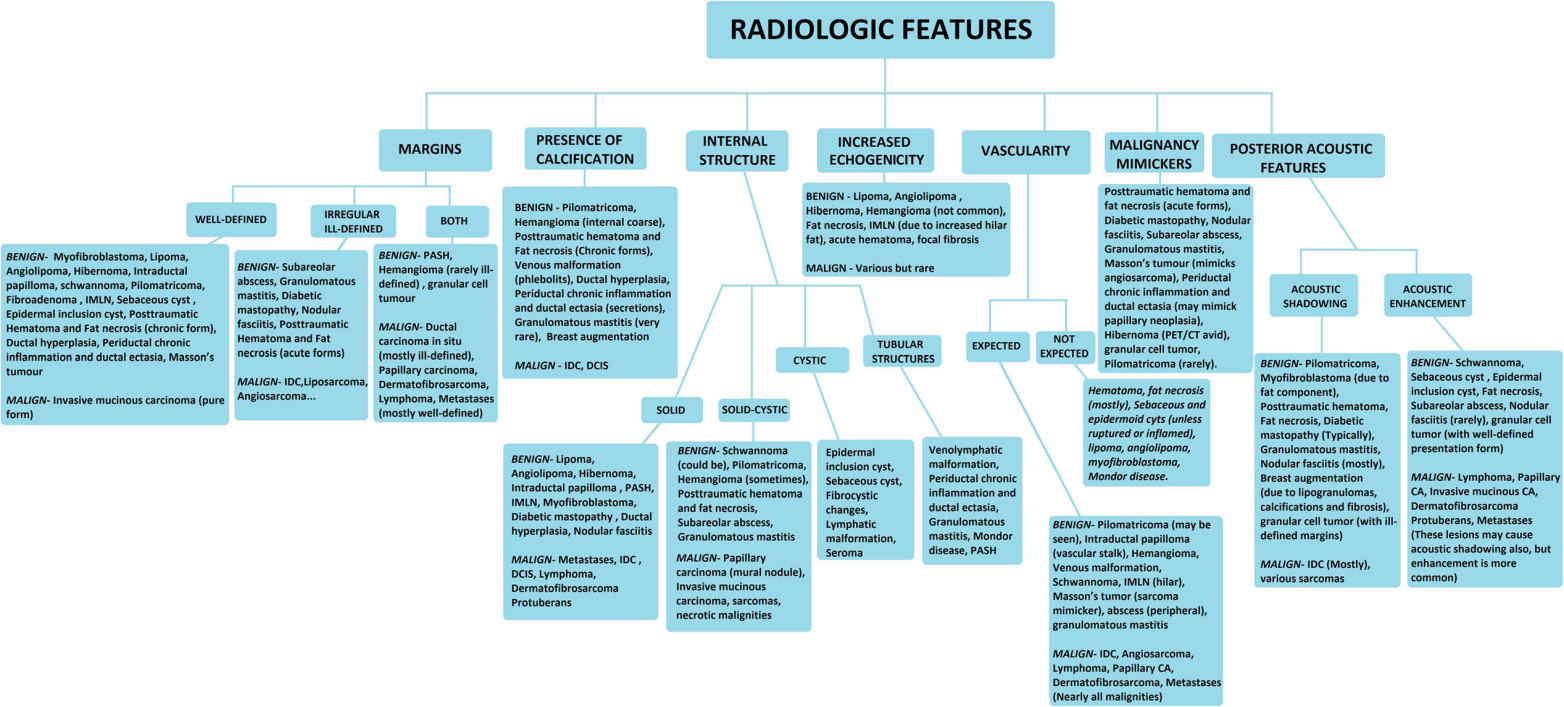
Classification based on “Radiologic features”

We can classify male breast lesions according to their known radiological features including margins, internal structure, presence of calcification and vascularity, hyperechogenicity, and posterior acoustic features. In general, benign neoplastic lesions have well-defined margins. On the other hand, not only most of the malign tumors but also the post-traumatic and inflammatory benign non-neoplastic lesions have ill-defined/irregular margins. Additionally, increased vascularity is more common in these groups. Increased echogenicity is less common in malign lesions. The presence of calcification and cystic/tubular internal structures can be important clues for narrowing the differential diagnoses. Furthermore, having knowledge of malignity-mimicker lesions and their clinical presentation can be helpful to the radiologist for being a part of proper patient management and facilitating diagnostic work-up. As a result, preventing unnecessary intervention and relieving patient anxiety can be accomplished with the knowledge of pitfalls which mimic malignity [6, 15]. For example, imaging appearances of acute hematoma/fat necrosis may mimic malignancy, but history of trauma to the lesion site is an important reason for recommending follow-up, and avoiding immediate biopsy. The comprehensive list of disease subgroups with their radiological features are shown in Fig. 8.

## Spectrum of disease

### Benign neoplastic breast lesions

#### Lipoma

Lipoma is the second most common benign male breast lesion which is composed of mature fat cells and is presented as asymptomatic or soft, non-tender palpable mass on physical examination. Radiologically, lipoma is seen as well-defined, subtle encapsulated, radiolucent lesion in mammography and avascular, oval-shaped, parallelly oriented, mild hyperechoic or iso-hypoechoic mass with thin echogenic capsule in US [3, 6, 15, 16].

#### Angiolipoma

Angiolipoma, which is a benign tumor formed by mature adipocytes, thin-walled vessels, and fibrin thrombi, is located typically anterior to the pectoralis fascia within fat tissue. Usual physical examination findings are painless, palpable, generally firm, mobile, and non-tender mass. Typical radiological findings are mass which is composed of mixed fat and soft-tissue densities in mammography and homogenous echogenic mass in US. Moreover, angiolipoma may not have any definite abnormality, in mammography. Differential diagnoses include acute hematoma, focal fibrosis, hemangioma, and malignancy [6, 19].

#### Hibernoma

It is slow-growing benign neoplastic lesion arising from brown adipose tissue. The physical examination findings are frequently mobile, painless, palpable, slow-growing mass. Hibernoma is an analogous of lipoma considering imaging findings. Sometimes, hibernoma may mimic malign lesions in different modalities. For example, in positron emission tomography-computed tomography (PET-CT) with fluorodeoxyglucose (FDG)-avidity, it may be impossible to distinguish it from well-differentiated liposarcomas reliably [20, 21].

#### Pseudoangiomatous stromal hyperplasia (PASH)

PASH is an uncommon stromal neoplasm composed of myofibroblasts with glandular hyperplasia within dense collagenous stroma. It is one of the benign conditions associated with gynecomastia, like as intraductal papilloma. PASH has two known forms: nodular mass-like and diffuse forms. Beside this, it could be seen incidentally within another lesion. Clinically, this benign lesion may be either asymptomatic or may present with focal palpable mass, especially in nodular form. Mammographic findings are “totally or partially circumscribed noncalcified breast mass” or “linear densities radiating from nipple similar to dendritic gynecomastia.” Sonographic imaging findings are “solid circumscribed, parallelly-oriented, hypoechoic mass with/without heterogeneity” or “retroareolar hypoechoic area with projecting tubular structures like gynecomastia” [7, 15, 16, 22].

#### Intraductal papilloma

**Fig. 9:**
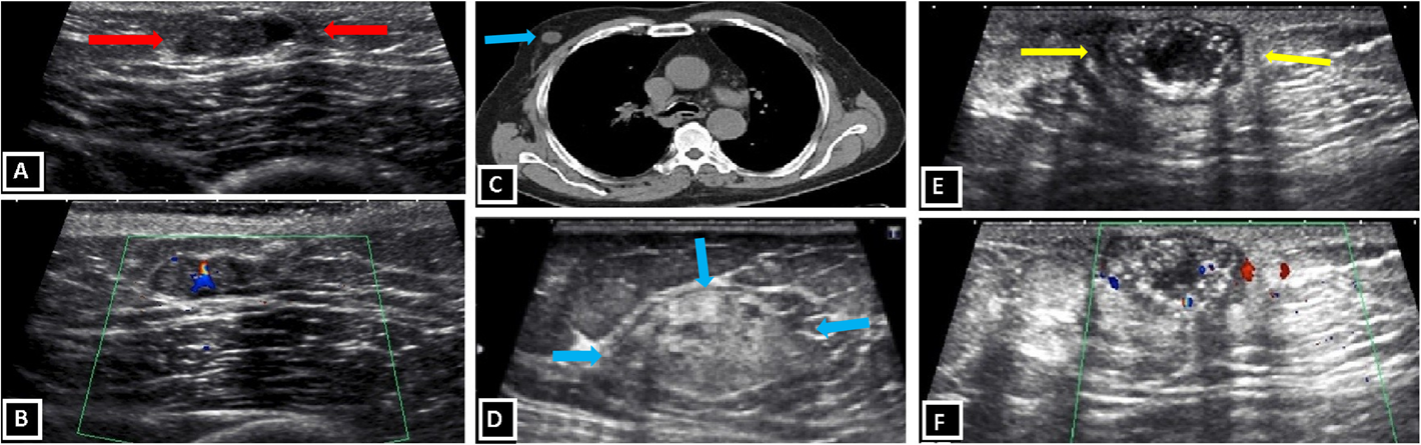
Benign neoplastic breast lesions. **a**, **b** Intraductal papilloma. Retroareolar well-defined solid nodule (red arrows) within a dilated duct is demonstrated in a 40-year-old patient with nipple discharge (**a**). Doppler ultrasound shows stalk of internal vascularity within the solid nodule (**b**). **c**, **d** Myofibroblastoma. Non-contrast CT scan displays well-defined round incidental mass (blue arrows) in right breast (**c**). US image shows circumscribed mass with mixed echogenicity and posterior acoustic shadowing due to internal fat content (**d**). **e**, **f** Pilomatricoma. Well-defined, lobulated complex mass with solid and cystic areas, hyperechoic spots, posterior acoustic shadowing (yellow arrows) and internal vascularity

It is a benign neoplasm of the intraductal epithelium with fibrovascular core. It may present with nipple discharge or palpable, painless, mobile, subareolar mass, on physical examination. Intraductal papilloma is, also, one of the benign conditions associated with gynecomastia. Imaging findings are discrete, dense mass in subareolar region with or without gynecomastia in mammography and subareolar, eccentric, elongated, well-defined, hypoechoic mass within dilated cystic ducts in US. “Stalk” of internal vascularity can be seen in Doppler US imaging. The differentiation of intraductal papilloma from papillary carcinoma is not possible with imaging alone; that is why the pathologic correlation is needed [6, 15, 16] (Fig. 9a, b).

#### Myofibroblastoma

Myofibroblastoma is an uncommon benign mesenchymal neoplastic lesion of the breast. It is more frequent in males than females and it affects mostly adult male population. Myofibroblastoma is seen as a mobile, well-defined, solid lump, on physical examination. Imaging findings identified in mammography are well-defined, encapsulated, heterogeneous tumor without microcalcifications. Sonographic findings are well-demarcated tumor, mixed echo pattern, and acoustic attenuation, probably, due to fat component [7, 23] (Fig. 9c, d).

#### Pilomatricoma

It is rare benign neoplastic skin tumor originating from piliferous follicles, which is also known as “calcifying epithelioma of Malherbe.” The most accused etiological factor is repeated skin trauma. Physical examination findings are non-tender, firm, palpable breast mass. The inflammatory phenomena with or without skin ulceration may also be seen. Radiologically, pilomatrixoma is seen as nodular opacity with punctate or heterogeneous calcifications, in mammography and mostly well-defined, circumscribed, parallel-oriented, heterogeneous mass composed of solid-cystic areas and hyperechoic calcifications with acoustic shadowing in US. Internal vascularity in Doppler imaging may also be seen [24, 25] (Fig. 9e, f).

#### Hemangioma

This rare vascular lesion of male breast is formed by proliferation of vascular channels lined by endothelial cells. It is located in either dermal or subcutaneous layers. Hemangioma may be asymptomatic or may present as palpable superficial breast mass, on physical examination. Imaging findings seen in mammography are well-defined, ovoid or lobulated, superficial mass with high density and internal coarse calcifications representing phleboliths. Sonographic findings are well-defined, ovoid or lobulated, superficial, non-hyperechoic lesion with an abrupt interface, with or without internal complex structure. Rarely, these vascular lesions could be seen as hyperechoic mass with indistinct margins [7, 26].

#### Schwannoma

This rare neoplasm arising from Schwann cells are seen as smooth, painless, soft mass, on physical examination. NF-2 related types have higher malignity potential than sporadic types. The mammographic findings are well-defined, round or oval mass. Well-defined, hypoechoic, solid mass with acoustic enhancement are typical sonographic findings. The other sonographic findings are complex heterogeneous mass with central cystic component and internal vascularity [6].

#### Granular cell tumor

Granular cell tumor is a rare benign neoplastic tumor arising from perineural Schwann cells of the peripheral nerves. Physical examination findings are painless, solid, non-tender nodule in the upper inner quadrant of the breast. These nodules can be multifocal. Imaging findings are non-specific and variable with a wide-range spectrum from “circumscribed mass with posterior acoustic enhancement” to “ill-defined mass with posterior acoustic shadowing.” They can mimic primary breast cancer [27].

### Benign non-neoplastic breast lesions

#### Epidermal inclusion cyst

It is the third most common benign lesion of the male breast, which arises from occluded hair follicles or sites of previous skin trauma. Physical examination findings are typically palpable, smooth, superficial lesion, and gland orifice which could be seen as a blackhead over mass. Furthermore, tenderness may be present in cases of rupture and surrounding inflammation. Epidermal inclusion cysts are seen as circumscribed, round or oval, superficial mass adjacent to skin with high density, in mammography. Tangential views can confirm the superficial location of the lesion. Sonographic findings are superficial (within dermis or subcutaneous fat), circumscribed, oval, hypoechoic mass, sometimes with “Onion skin” appearance which represents alternating concentric hypo and hyperechoic rings due to the accumulation of lamellated keratin. Rarely fistula tract can be seen between lesion and skin, in US [3, 15].

#### Sebaceous cyst

Sebaceous cyst is benign intradermal lesion formed by obstruction of sebaceous gland. It is generally smaller than epidermal inclusion cyst. Sebaceous cyst is one of the “don’t touch” lesions when suspected because biopsy may induce inflammatory process in the surrounding breast tissues. Clinically, sebaceous cyst is seen as palpable, superficial breast lump. Inflammatory changes may be present in cases of rupture or surrounding inflammation. The imaging findings are similar to epidermal inclusion cyst, and mostly indistinguishable with imaging alone. The “claw sign,” which refers to wrapping of dermal tissue around the lesion like a claw, may be seen in US and could be helpful for differentiation from superficial breast parenchymal lesions. In Doppler US imaging, they do not have internal vascularization with the exception of peripheral hypervascularization which is seen in inflamed or ruptured cases [6, 7, 28].

#### Post-traumatic hematoma and fat necrosis

**Fig. 10:**
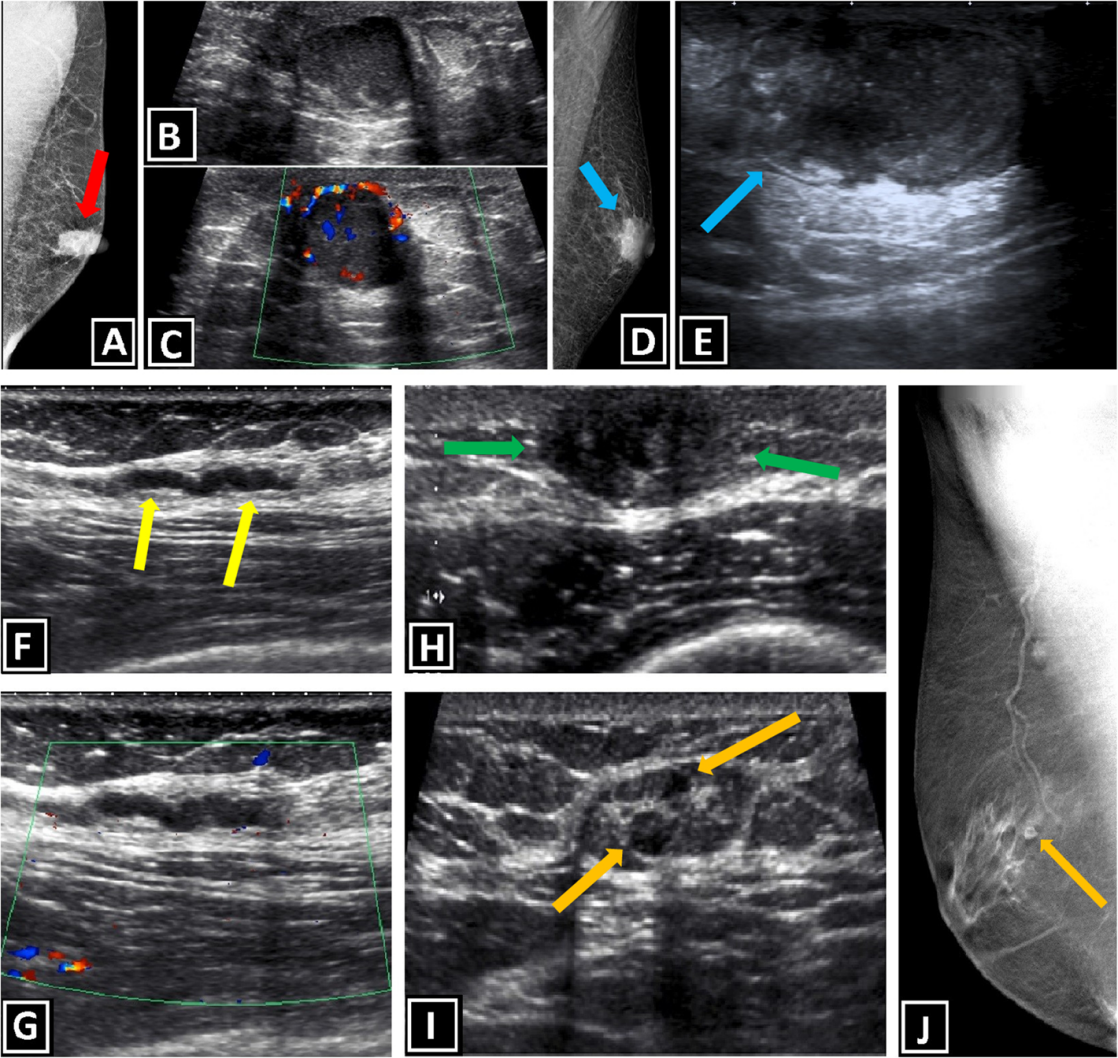
Benign non-neoplastic breast lesions. **a**–**c** Fat necrosis and periductal mastitis. Mammography of a 44-year-old man with the complaint of left retroareolar pain shows circumscribed retroareolar lesion with high density (red arrow, **a**). Well-defined, round hypoechoic lesion with internal and peripheral vascularity is seen in US (**b**, **c**). Biopsy reveals the diagnosis. **d**, **e** Subareolar abscess secondary to ruptured folliculitis. Mammography of 64-year-old man with retroareolar mass and skin erythema shows subareolar lesion with spicular margins and high density (blue arrow, **d**). US for exclusion of malignancy reveals heterogeneous, hypoechoic mass which has cystic component with internal echogenities and irregular walls (blue arrow, **e**). Increased vascularity in surrounding tissues is also present (not shown). Pathologic examination of mastectomy material reveals the diagnosis. **f**, **g** Mondor’s disease. US and Doppler US examinations of 32-year-old patient with localized right breast pain show “beaded tubular vascular structure with irregular walls and minimally increased peripheral echogenicity, without apparent blood flow” at the symptomatic area (yellow arrows, **f**). **h** Duct hyperplasia. Retroareolar well-defined hypoechoic lesion is shown in a 41-year-old man with nipple discharge (green arrows). **i** Two small intramammary lymph nodes with circumscribed margin and round shape are shown (orange arrows). **j** Breast tomosynthesis of another patient reveals gynecomastia accompanied by intramammarian lymph node with round shape and radiolucent fatty hilus (orange arrow)

Fat necrosis is formed by necrotic adipocytes surrounded by lipid-laden macrophages. Hematoma is a localized collection of blood and blood products outside the blood vessels. These are benign processes, generally related to trauma or breast surgery. The clinical presentation is usually new palpable breast lesion which could be painful. The history of trauma, surgery, or anticoagulation is generally present. Mammographic imaging findings are related with the phase of lesion. Acute forms can mimic malignity or abscess due to ill-defined mass with skin thickening, and trabecular prominence. Chronic forms turn into more discrete lesions which may contain fat-fluid levels. Distortions, dystrophic calcifications, and persistent well-defined masses are common mammographic findings during this stage. Sonographic findings are avascular, circumscribed, complex, heterogeneous mass with possible fluid-fluid levels, and internal septation [3, 6] (Fig. 10a–c).

#### Seroma

This lesion usually develops after surgery or breast trauma, and it may be seen as painful mass on physical examination. An anechoic fluid collection with or without internal septation is the initial imaging finding. During maturation, a collection with thick, nodular margin can be seen [29].

#### Nodular fasciitis

It is one of the benign mesenchymal tumors characterized by pseudoneoplastic reactive proliferation of myofibroblast. The breast involvement is rare. The etiology is unclear while local trauma is accused for triggering reactive proliferation by the majority of authors. Physical examination findings are hard, well-defined, mobile, painless, palpable mass which shows rapid growth with sudden onset, generally, and may mimic malignancy. Radiologically, nodular fasciitis is seen as ill-defined density with irregular margins, without calcifications, in mammography and hypoechoic, heterogeneous, solid mass with irregular margins, without prominent vascularization, in US [6, 30, 31].

#### Diabetic mastopathy

This rare breast disease is characterized by stromal sclerosis, and dense lymphocytic infiltration containing fibroinflammatory process. It is typically seen in patients with long-term type 1 diabetes mellitus. Diabetic mastopathy may mimic malignity both radiologically and clinically. That is why, the history of long-standing diabetes mellitus is crucial for diagnosis. Generally, systemic complications of diabetes coexist with diabetic mastopathy due to long-term disease. There is usually large, painless, firm, palpable breast mass with multicentric or bilateral involvement on physical examination. Radiologically, ill-defined mass or asymmetric densities are usual mammographic findings. Sonographic findings are hypoechoic mass or masses with irregular margins, and posterior acoustic shadowing [6].

#### Periductal chronic inflammation and ductal ectasia

It is a rare disease of the male breast characterized by chronic inflammation, and fibrosis around dilated ducts which may contain debris. It can mimic malignity due to its clinicopathologic properties. Nipple discharge, subareolar tender breast mass, and nipple retraction can be seen on physical examination. Additionally, it could be complicated with subareolar abscess or fistula formation. Common mammographic findings are subareolar ductal dilatation with intraductal calcified secretions, and coexistent mass-like opacities in subareolar location. Retroareolar dilated ducts which contain internal debris is seen as tubular structures in US. So the differentiation from papilloma or papillary carcinoma may be required. Subareolar mass is another sonographic finding which could be present [32, 33] (Fig. 10a–c).

#### Subareolar abscess

It is also called as “Zuska’s disease,” and it is characterized by subcutaneous abscess in subareolar location due to aseptic inflammation caused by squamous metaplasia which is secondary to obstruction of lactiferous ducts. Physical examination findings are mastalgia, nipple discharge, inflammatory signs, tender subareolar breast lump. Fistulas can be seen in chronic cases. Diagnosis usually requires the knowledge of clinical history and physical examination. Because imaging findings may mimic malignity or gynecomastia. Mammography is hard to obtain due to pain with breast compression. When it is done, imaging findings like skin thickening, distortion, and ill-defined subareolar mass accompanied by trabecular thickening could be seen. Sonographic imaging findings present as “heterogenous, irregular, hypoechoic mass” or “fluid collection with internal echoes, and irregular walls.” The increased vascularity in surrounding tissues, thickening of skin due to inflammation, and fistulous tract formation may also be present [6, 34, 35] (Fig. 10d, e).

#### Idiopathic and specific granulomatous mastitis

These are very rare chronic inflammatory male breast diseases characterized by non-caseating granulomatous lobulitis. They may mimic breast cancer both clinically and radiologically. The etiology of idiopathic granulomatous mastitis is not clearly known. While specific granulomatous mastitis usually has certain etiology like tuberculosis, Wegener’s granulomatosis, vasculitis, sarcoidosis, foreign body reaction, syphilis, corynebacterial infection, and fungal and parasitic infections. Identification of etiology is important for both prognosis and treatment. Clinico-radiologic findings of these diseases are similar to each other. Physical examination findings are subareolar, firm, painless, or tender breast mass. Inflammatory symptoms may be present, and they are usually resistant to empiric antibiotic therapy without relief. Mammographic imaging findings are ill-defined opacities, parenchymal distortion, and masses with non-distinct margins. Ill-defined hypoechoic heterogeneous lesions which may show tubular extensions, and increased vascularization accompanied by peripheral distortion are known sonographic findings [36–38].

#### Mondor’s disease

It is rare self-limiting breast lesion characterized by superficial thrombophlebitis of the anterior chest wall including the superior epigastric, lateral thoracic, thoracoepigastric veins. The exact etiology or the pathogenesis is not known. The most accused factors are direct trauma to the veins, invasive procedures like surgery and biopsy, inflammatory process, and breast cancer. The expected physical examination finding is initially soft and red cord-like structure which later turns into painful and rigid breast mass. Radiologically, Mondor’s disease is seen as dilated tubular density beneath the skin in mammography and tubular, beaded hypoechoic or anechoic structure with thick hyperechoic wall in US. There is usually no flow in this tubular structure in Doppler US scan [8, 39] (Fig. 10f, g).

#### Masson’s tumor

This rare breast lesion is caused by organization and recanalization of preexisting thrombus with benign vascular proliferation. It is also known as papillary endothelial hyperplasia (PEH). During the organization of thrombus, anastomosing vascular channels develop and lesion becomes prominently vascular. Because of this, angiosarcoma, which is the most frequent vascular malign tumor of the breast, is the important differential diagnosis and it must be excluded. However, differentiation of Masson’s tumor from angiosarcoma is not possible always, even pathologically. The imaging findings are non-specific [40].

#### Ductal hyperplasia

Ductal hyperplasia is defined as intraductal epithelial cell proliferation with or without atypia. It could be seen incidentally in gynecomastia. Although, physical examination findings are non-specific, nipple discharge or palpable mass may be seen. Imaging findings of ductal hyperplasia have been rarely described. The cluster of amorphous microcalcification may be seen in mammography, especially in case of atypical ductal hyperplasia. Well-defined nodular lesion or area of distortion may be present in US [41–43] (Fig. 10h).

#### Venous malformation

It is the most common type of vascular malformation in the male breast, consisting of multiple ectatic thin-walled vessels lined by endothelial cells. The slow internal flow without shunting is a characteristic feature. Common physical examination findings are unilateral, painless, chronic breast enlargement. Venous malformation is seen as multiple tubular densities with or without phleboliths in mammography, while multiple tubular, anechoic spaces with internal vascularity in US. The slow venous flow in Doppler US is a very important finding for differential diagnosis, since lack of flow suggests the lymphatic malformation. The phleboliths and thrombosis are other findings that can be detected sonographically [6].

#### Intramammary lymph node (IMLN)

IMLNs can be anywhere but generally located in upper-outer quadrant, and the most common physical examination findings are palpable, mobile breast mass. Imaging findings of the IMLN are “oval or reniform lesion with radiolucent fatty hilus and denser peripheral cortex” in mammography and “echogenic hilus with thin (< 2–3 mm), homogenous, hypoechoic cortex” in US. Sonographically, short-axis dimension > 10 mm, round or irregular shape and cortical thickening (> 2–3 mm) is considered as abnormal [6] (Fig. 10i, j).

#### Breast augmentation

Augmented breasts have diffuse gynecomastia and female breast appearance due to hormonal therapy. “Surgically placed breast implants” and “direct injection of viscous fluids: mineral oils like industrial silicone or paraffin” are other alternatives, in transexual patients. The mineral-oil injection may lead to inflammation and necrosis of breast. Long-term complication of the mineral-oil injection is known as “sclerosing lipogranulomatosis.” Physical examination findings are inflammation, fibrosis, and palpable mass-like lesions (sclerosing lipogranulomas). Mammographic findings are various such as "Female breast" appearance with extensive microcalcifications and/or coarse calcifications, parenchymal opacities and distortions. Diffuse gynecomastia, breast implants, calcifications with acoustic shadowing, distortions due to fibrosis or inflammatory breast masses can be seen in US imaging [6, 44].

### Malign breast lesions

#### Invasive ductal carcinoma (IDC)

**Fig. 11:**
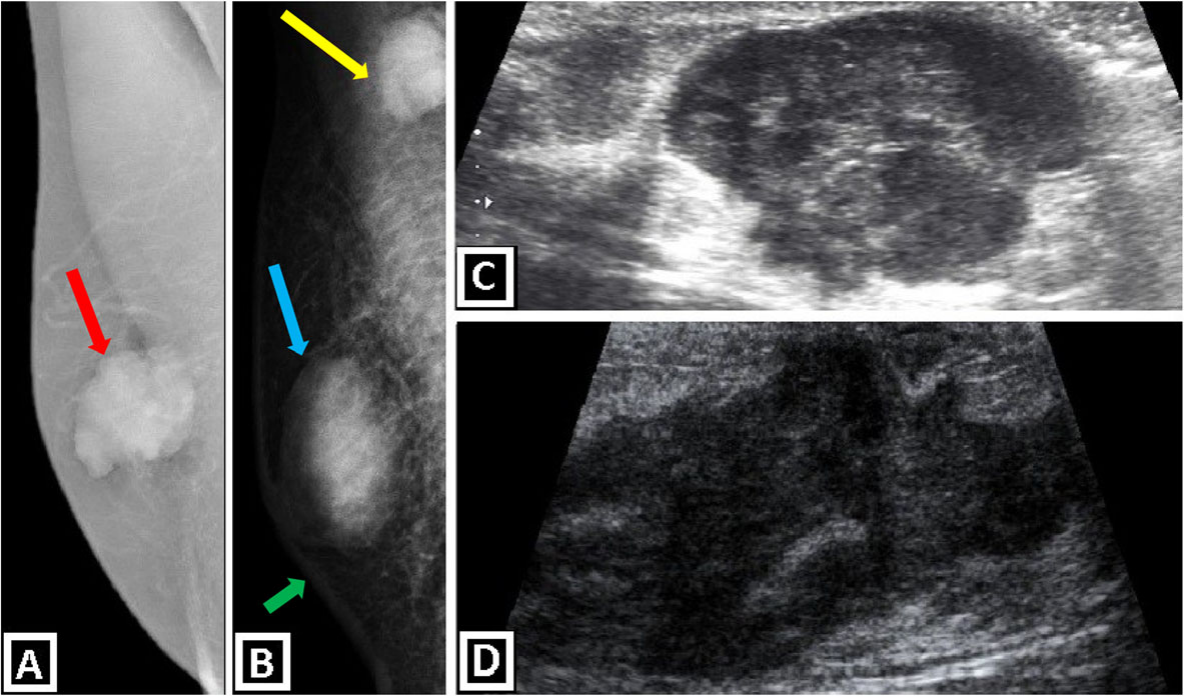
Mammography and US images show primary male breast cancer. **a**, **b** Medio-lateral oblique mammograms show invasive ductal carcinoma of the breast. Lobulated subareolar masses with high density, without microcalcification are seen (red and blue arrows). **a** Grade 2 invasive ductal carcinoma. **b** Grade 3 invasive ductal carcinoma with axillary lymph node metastasis (yellow arrow) and skin thickening (green arrow). US images of grade 2 (**c**) and grade 3 (**d**) invasive ductal carcinomas reveal irregular shape and indistinct margins of the masses

It is the most common primary malign neoplastic lesion of male breast, and almost 80% of all cases belong to IDC “not otherwise specified type.” On physical examination, these lesions are seen as hard, painless, palpable mass with secondary features such as nipple retraction, skin thickening, palpable axillary LAP. The latter one accompanies IDC in 50% of the cases. The discrete mass appearance and secondary features are important clues for malignity. Bilateral mammography is recommended in case of malignity suspicion, because underlying risk factors may cause the development of contralateral breast malignity. In mammography, IDC is seen as radiodense, irregular, retroareolar masses with spiculated, lobulated or microlobulated margins. The incidence of microcalcifications is lower than female breast cancer. Retroareolar, non-parallel, hypoechoic mass with irregular borders and variable vascularity are well-known US findings. Accompanying secondary findings can also be seen in US [3, 4, 7, 9, 15] (Fig. 11).

#### Ductal carcinoma in situ (DCIS)

It is the second most common subtype of male breast cancer and responsible for 5% of all cases. The pure form of DCIS is rarely present due to the very high rate of coexistence with invasive carcinoma. Palpable mass and/or bloody nipple discharge is frequently seen in physical examination. DCIS is seen as pleomorphic microcalcifications which represent in situ component of tumor cells, in mammography. However, parenchymal opacity or distortion may, also, be seen if invasive carcinoma accompanies. The findings on US are non-specific, but coexistent IDC foci may be seen as mass or distortion [6, 7, 9].

#### Papillary carcinoma

Papillary carcinoma is more common in men than women (2:1) accounting for 2.5–5% of male breast cancers and presents with palpable subareolar mass. Mammographic findings are circumscribed, oval, lobulated or irregular, subareolar mass. Ill-defined borders point out infiltrative component. Typical sonographic appearance is complex heterogeneous mass formed by solid and cystic components. “Solid mural nodule in complex mass” or “pure solid mass” are other possible findings [3, 7].

#### Invasive mucinous carcinoma

IMC is a histological variant of breast carcinoma characterized by the extracellular mucin component surrounding neoplastic cells. It accounts for 1% of male breast cancers. Pure and mixed forms are present. Physical examination findings are mostly non-specific. Palpable hard subareolar mass may be seen. Imaging findings are, also, non-specific. Pure form may be confused with benign breast lesions, because the characteristic mammographic finding of pure form is round and well-defined opacity, and the expected sonographic finding is well-defined subareolar iso-hypoechoic lesion [7].

#### Dermatofibrosarcoma protuberans

This uncommon lesion, which is locally aggressive, slow-growing fibrous tumor involving the dermal layer of the skin with intermediate-to-low grade malignancy, is more frequent in males than females. Due to its local aggressive characteristics, local recurrence rate after the surgery is very high. It presents as firm, painless, slow-growing, generally mobile, palpable breast lump, on physical examination. Mammographic findings are ill-defined radiodense breast mass without fat or calcification. An ovoid lesion with lobulated or microlobulated margins located in dermis or subcutaneous tissue is sonographic appearance. Mixed internal echogenicity and hypervascularization can also be seen in US [15, 45, 46].

#### Paget’s disease

This disease is characterized by skin changes of nipple and areola, such as eczematous scaling, skin erosions, and ulceration associated with the possible ductal spread of breast cancer. It is mostly a clinical diagnosis, and imaging findings are non-diagnostic. However, coexistent DCIS and/or invasive carcinoma may be present, and their imaging findings can be seen. After suspicion “based on history and physical examination,” biopsy confirms the diagnosis [7].

#### Lymphoma

**Fig. 12:**
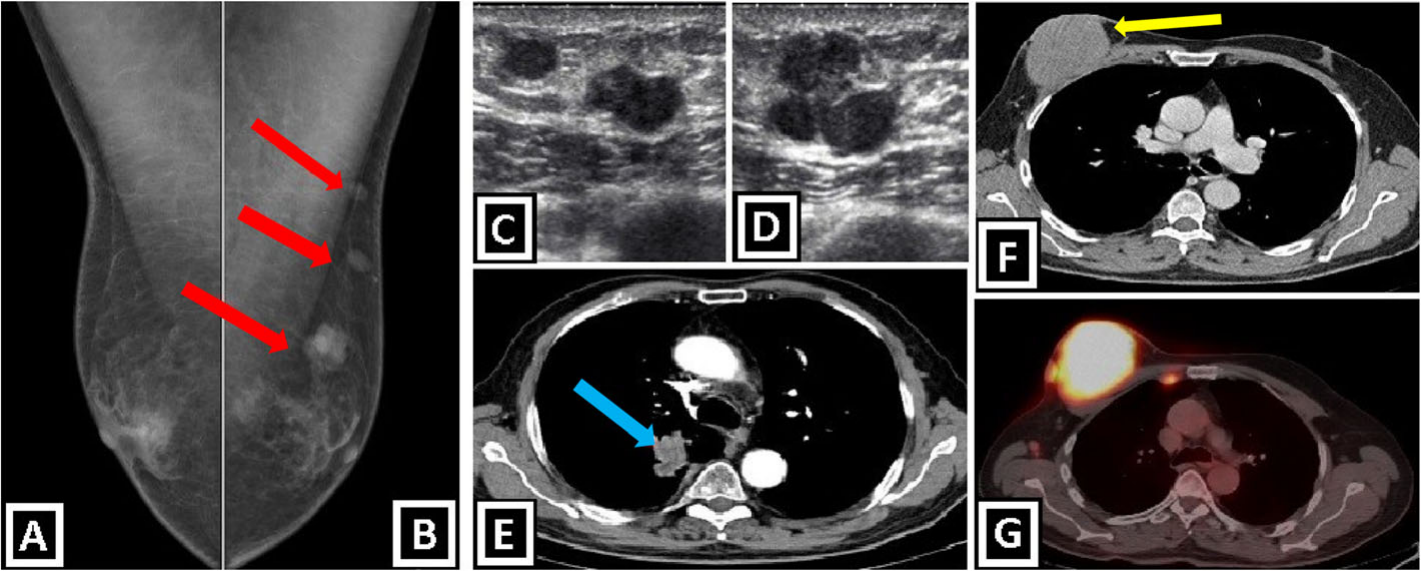
Secondary malignant breast masses in males. **a**–**e** Radiological images of a 58-year-old man with lung adenocarcinoma. **a**, **b** Bilateral medio-lateral oblique mammograms show bilateral gynecomastia and three round masses in left breast (red arrows). **c**, **d** US images demonstrate macrolobulated round hypoechoic metastatic masses. **e** Contrast-enhanced axial CT image shows lung adenocarcinoma in left lung (blue arrow). **f**, **g** Breast involvement of diffuse large B cell lymphoma in a 63-year-old patient. Contrast enhanced axial CT image shows a circumscribed mass within left breast (**f**). PET-CT scan reveals FDG-avid masses which are consistent with lymphamatous infiltration located in not only right breast, but also right internal mammarian and axillary lymph node stations (**g**)

Lymphoma involvement of male breast may be primary or secondary. Secondary cases mostly related to non-Hodgkin B cell lymphoma involvement. Physical examination findings are enlarged axillary lymph nodes, single or multiple palpable breast masses. The history of lymphoma is an important clue for the diagnosis. Mammographic imaging findings are “single or multiple, circumscribed or ill-defined lesions” and “multiple, enlarged, circumscribed, oval or lobular, radiodense axillary lymph nodes without apparent radiolucent fatty hilus”. Findings in US imaging are “circumscribed or irregular, hypoechoic, solid mass” or “masses accompanied by enlarged axillary lymph nodes with irregularly thickened cortex and without normal echogenic fatty hilus structure” [1, 3, 7, 15] (Fig. 12f, g).

#### Metastases

Breast metastases from extramammarian malignancies are rare, and they point out late-stage disseminated disease. The presence of multiple bilateral lesions and subcutaneous fat tissue location are supportive features for metastases, while “unilateral lesion” and “glandular breast tissue involvement” support primary breast cancer. Regarding breast metastases, skin or nipple retraction is not expected in contrast to primary breast cancer. Multiple bilateral palpable solid masses with a known primary malignancy (such as prostate cancer, lung cancer, gastric cancer, colorectal cancer, melanoma, or sarcoma) suggest the possibility of breast metastases. Prostate cancer is accepted as the most frequent primary site for breast metastasis. Mammographic imaging findings are single or multiple (mostly), round or lobulated masses with circumscribed or ill-defined (less expected) borders. The diffuse skin thickening may, also, be seen. Calcifications and spiculated borders are not expected findings opposite to primary breast cancer. Main imaging findings in US are hypoechoic, circumscribed masses. Increased internal vascularity and posterior acoustic enhancement are other features that may be present [1, 4, 18] (Fig. 12a–e).

## Conclusion

Understanding the histoanatomic differences between male and female breasts has utmost importance for insighting the diversities in radiological appearance, diagnostic algorithm, biopsy procedure, and malignancy characteristics, between the two sexes. Majority of breast lumps in male patients are benign, and the most common reason is gynecomastia. Except for gynecomastia, male breast has wide-range spectrum of pathologies and hosts many lesions which can be classified according to various radiologic and histopathologic characteristics. Familiarity with the imaging findings, histopathological properties, and presentation features of various benign and malignant male breast lesions allows correct imaging interpretation which can facilitate “convenient patient management,” “reaching early and confident diagnosis,” and “avoiding unnecessary interventions”.

## Data Availability

Data sharing is not applicable to this article as no datasets were generated or analyzed during the current study.

## Abbreviations

DCIS: Ductal carcinoma in situ
FDG: Fluorodeoxyglucose
IDC: Invasive ductal carcinoma
ILC: Invasive lobular carcinoma
IMLN: Intramammary lymph node
LAP: Lymphadenopathy
LCIS: Lobular carcinoma in situ
MRI: Magnetic resonance imaging
NF: Neurofibromatosis
PASH: Pseudoangiomatous stromal hyperplasia
PEH: Papillary endothelial hyperplasia
PET-CT: Positron emission tomography-computed tomography
TDLU: Terminal ductal-lobular unit
US: Ultrasound

## References

[1] Iuanow E, Kettler M, Slanetz PJ (2011). Spectrum of disease in the male breast. AJR Am J Roentgenol.

[2] Omene C, Tiersten A (2010) The differences between male and female breast cancer. In: Legato MJ (Ed) Principles of gender-specific medicine. Elsevier, pp 459–472. 10.1016/b978-0-12-374271-1.00042-3

[3] Chau A, Jafarian N, Rosa M (2016). Male breast: clinical and imaging evaluations of benign and malignant entities with histologic correlation. Am J Med.

[4] Charlot M, Béatrix O, Chateau F, Dubuisson J, Golfier F, Valette PJ, Réty F (2013). Pathologies of the male breast. Diagn Interv Imaging.

[5] Senger JL, Adams SJ, Kanthan R (2017) Invasive lobular carcinoma of the male breast- a systematic review with an illustrative case study. Breast Cancer (Dove Med Press) 9:337-345. 10.2147/BCTT.S126341. 10.2147/BCTT.S126341 PMC5439541 28553141

[6] Nguyen C, Kettler MD, Swirsky ME, Miller VI, Scott C, Krause R, Hadro JA (2013). Male breast disease: pictorial review with radiologic-pathologic correlation. Radiographics..

[7] Yen PP, Sinha N, Barnes PJ, Butt R, Iles S (2015). Benign and malignant male breast diseases: radiologic and pathologic correlation. Can Assoc Radiol J.

[8] Draghi F, Tarantino CC, Madonia L, Ferrozzi G (2011). Ultrasonography of the male breast. J Ultrasound.

[9] Yuan WH, Li AF, Chou YH, Hsu HC, Chen YY (2018) Clinical and ultrasonographic features of male breast tumors: a retrospective analysis. PLoS One 13(3):e0194651. 10.1371/journal.pone.0194651 10.1371/journal.pone.0194651 PMC5860767 29558507

[10] Shin K, Martaindale S, Whitman GJ (2018) Male breast magnetic resonance imaging: when is it helpful? Our experience over the last decade. Curr Probl Diagn Radiol 48(3):196–203. 10.1067/j.cpradiol.2018.01.002 29454681

[11] Shaw A, Smith B, Howlett D (2015). Male breast carcinoma and the use of MRI. Radiol Case Rep.

[12] Kipling M, Ralph JEM, Callanan K (2014) Psychological impact of male breast disorders: literature review and survey results. Breast Care (Basel) 9(1):4–4. 10.1159/000358751 10.1159/000358751 PMC3995375 24803884

[13] Mustapha Z, Haliru MA, Ismail A, Yakubu SD (2016) Pictorial essay: a retrospective review of male breast diseases in Maiduguri and Kano, Nigeria. West Afr J Radiol 23:107-112. 10.4103/1115-3474.187969.

[14] Mainiero MB, Lourenco AP, Barke LD, Argus AD, Bailey L, Carkaci S, D'Orsi C, Green ED, Holley SO, Jokich PM, Lee SJ, Mahoney MC, Moy L, Slanetz PJ, Trikha S, Yepes MM, Newell MS (2015). ACR appropriateness criteria evaluation of the symptomatic male breast. J Am Coll Radiol.

[15] Chen L, Chantra PK, Larsen LH et al (2006) Imaging characteristics of malignant lesions of the male breast. Radiographics 26(4):993–1006. 10.1148/rg.264055116 10.1148/rg.264055116 16844928

[16] Yitta S, Singer CI, Toth HB, Mercado CL (2010). Sonographic appearances of benign and malignant male breast disease with mammographic and pathologic correlation. J Ultrasound Med.

[17] Friedrich RE, Hagel C, Mautner VF (2015) Unilateral gynaecomastia in a 16-monthold boy with neurofibromatosis type 1 - case report and brief review of theliterature. GMS Interdiscip Plast Reconstr Surg DGPW 4: Doc11. 10.3205/iprs000070. 10.3205/iprs000070 PMC4670967 26668786

[18] Genç B, Solak A, Sahin N, Gülşen A (2013). Metastasis to the male breast from squamous cell lung carcinoma. Case Rep Oncol Med.

[19] Weinstein SP, Conant EF, Acs G (2003). Case 59: Angiolipoma of the breast. Radiology..

[20] DeRosa DC, Lim RB, Lin-Hurtubise K, Johnson EA (2012). Symptomatic hibernoma: a rare soft tissue tumor. Hawaii J Med Public Health.

[21] Daubner D, Spieth S, Pablik J, Zöphel K, Paulus T, Laniado M (2015). Hibernoma—two patients with a rare lipoid soft-tissue tumour. BMC Med Imaging.

[22] Mizutou A, Nakashima K, Moriya T (2015). Large pseudoangiomatous stromal hyperplasia complicated with gynecomastia and lobular differentiation in a male breast. Springerplus..

[23] Mele M, Jensen V, Wronecki A, Lelkaitis G (2011). Myofibroblastoma of the breast: case report and literature review. Int J Surg Case Rep.

[24] AlSharif S, Meguerditchian A, Omeroglu A, Lamarre P, Altinel G, Mesurolle B (2015). Pilomatricoma of the male breast: sonographic mammographic MRI features with pathologic correlation. Clin Imaging.

[25] Nori J, Abdulcadir D, Giannotti E, Calabrese M (2013). Pilomatrixoma of the breast, a rare lesion simulating breast cancer: a case report. J Radiol Case Rep.

[26] Mesurolle B, Sygal V, Lalonde L, Lisbona A, Dufresne MP, Gagnon JH, Kao E (2008). Sonographic and mammographic appearances of breast hemangioma. AJR Am J Roentgenol.

[27] Jagannathan DM (2016). Benign granular-cell tumor of the breast: case report and literature review. Radiol Case Rep.

[28] Giess CS, Raza S, Birdwell RL (2011). Distinguishing breast skin lesions from superficial breast parenchymal lesions: diagnostic criteria, imaging characteristics, and pitfalls. Radiographics..

[29] Oliveira LT, Aguiar SS, Bender PF, Bergmann A, Thuler LC (2017) Men have a higher incidence of seroma after breast cancer surgery. Asian Pac J Cancer Prev 18(5):1423–1427. doi: 10.22034/APJCP.2017.18.5.1423. 10.22034/APJCP.2017.18.5.1423 PMC5555557 28612597

[30] Squillaci S, Tallarigo F, Patarino R, Bisceglia M (2007). Nodular fasciitis of the male breast: a case report. Int J Surg Pathol.

[31] Paliogiannis P, Cossu A, Palmieri G et al (2016) Breast nodular fasciitis: a comprehensive review. Breast Care (Basel) 11(4):270–274. 10.1159/000448185 10.1159/000448185 PMC5040882 27721715

[32] Ashworth MT, Corcoran GD, Haqqani MT (1985). Periductal mastitis and mammary duct ectasia in a male. Postgrad Med J.

[33] Zhang Y, Zhou Y, Mao F, Guan J, Sun Q (2018) Clinical characteristics, classification and surgical treatment of periductal mastitis. J Thorac Dis 10(4):2420–2427. 10.21037/jtd.2018.04.22. 10.21037/jtd.2018.04.22 PMC5949503 29850148

[34] Kazama T, Tabei I, Sekine C, Funamizu N, Onda S, Okamoto T, Takeyama H, Morikawa T (2017). Subareolar breast abscess in male patients: a report of two patients with a literature review. Surg Case Rep.

[35] Johnson SP, Kaoutzanis C, Schaub GA (2014) Male Zuska’s disease. BMJ Case Rep. 10.1136/bcr-2013-201922. 10.1136/bcr-2013-201922 PMC3987215 24706699

[36] Al Manasra AR, Al-Hurani MF (2016). Granulomatous mastitis: a rare cause of male breast lump. Case Rep Oncol.

[37] Reddy KM, Meyer CE, Nakdjevani A, Shrotria S (2005). Idiopathic granulomatous mastitis in the male breast. Breast J.

[38] Korkut E, Akcay MN, Karadeniz E, Subasi ID, Gursan N (2015). Granulomatous mastitis: a ten-year experience at a university hospital. Eurasian J Med.

[39] Amano M, Shimizu T (2018). Mondor’s disease: a review of the literature. Intern Med.

[40] Branton PA, Lininger R, Tavassoli FA (2003). Papillary endothelial hyperplasia of the breast: the great impostor for angiosarcoma. Int J Surg Pathol.

[41] Prasad V, M King J, McLeay W, Raymond W, Cooter RD (2005). Bilateral atypical ductal hyperplasia, an incidental finding in gynaecomastia--case report and literatüre review. Breast..

[42] Latronico A, Nicosia L, Faggian A, Abbate F, Penco S, Bozzini A, Cannataci C, Mazzarol G, Cassano E (2018). Atypical ductal hyperplasia: our experience in the management and long term clinical follow-up in 71 patients. Breast..

[43] Dickerson E, Budway R, Surampudi R, Tabor E (2010). Mammary duct ectasia in a man with liver disease, end stage renal failure, and adjacent arteriovenous fistula. J Radiol Case Rep.

[44] Hazani R, Engineer N (2008). Surreptitious injection of mineral oil: a case report of sclerosing lipogranulomatosis. Ann Plast Surg.

[45] Mirza TI, Akhtar K, Abbas HB, Sameena M, Tahir F, Khan S, Bhutto AUR (2011). Dermatofibrosarcoma protuberans male breast: a case report. Oman Med J.

[46] Saikia BK, Das I, Mandal GK (2016). Fibrosarcomatous change in the background of dermatofibrosarcoma protuberans in male breast: study of a rare case and review of the entity. J Midlife Health.

